# Effects of a Phytogenic Water Supplement on Selected Cultivable Bacterial Populations and Intestinal Histomorphology in Ross 308 Broiler Chickens Challenged with *Salmonella* Typhimurium

**DOI:** 10.3390/life16081341

**Published:** 2026-08-16

**Authors:** Cristina-Elena Horhogea, Ivona Popovici, Geta Pavel, Carmen Solcan, Lenuța Fotea, Mariana Grecu, Erzsébet Varga, Ibolya Fülöp, Dragoș-Constantin Aniță, Cristina-Mihaela Rîmbu

**Affiliations:** 1 Department of Public Health, Faculty of Veterinary Medicine, Ion Ionescu de la Brad Iasi University of Life Sciences, 700489 Iași, Romania; cristina.horhogea@iuls.ro; 2 Department of Preclinics, Faculty of Veterinary Medicine, Ion Ionescu de la Brad Iasi University of Life Sciences, 700489 Iași, Romania; geta.pavel@iuls.ro (G.P.); carmen.solcan@iuls.ro (C.S.); mariana.grecu@iuls.ro (M.G.); dragos.anita@iuls.ro (D.-C.A.); 3 Department of Management of Animal Resources and Technologies, Faculty of Food ans Animal Sciences, Ion Ionescu de la Brad Iasi University of Life Sciences, 700490 Iași, Romania; lenuta.fotea@iuls.ro; 4 Department of Pharmacognosy and Phytotherapy, Faculty of Pharmacy, University of Medicine, Pharmacy, Science, and Technology of Târgu Mureș, 540142 Târgu Mureș, Romania; erzsebet.varga@umfst.ro; 5 Department of Toxicology and Biopharmacy, Faculty of Pharmacy, University of Medicine, Pharmacy, Science, and Technology of Târgu Mureș, 540142 Târgu Mureș, Romania; ibolya.fulop@umfst.ro; 6 Center of Advanced Research for Emerging Diseases, Zoonoses and Food Safety (ROVETEMERG), 700489 Iași, Romania

**Keywords:** phytogenic formulation, *Salmonella* Typhimurium, Ross 308 broiler chickens, intestinal cultivable bacterial populations, intestinal histomorphometry, immunohistochemistry

## Abstract

Increasing antimicrobial resistance and restrictions on antibiotic use in poultry have drawn attention to phytogenic products as a complementary approach for controlling enteric bacterial infections. The aim of this study was to evaluate the effects of a phytogenic product (Herba Top Salmonella, HTS) on selected cultivable intestinal bacterial populations, intestinal histomorphology and tissue distribution of *Salmonella* Typhimurium in experimentally infected broiler chickens. A total of sixty one-day-old Ross 308 broilers were randomly assigned to six experimental groups (10 birds/group; two pens/group, five birds/pen, with the pen considered the experimental unit. Birds in three groups were experimentally challenged with *Salmonella enterica* subsp. *enterica* serovar Typhimurium, and HTS was administered in drinking water for 14 consecutive days at two nominal concentrations (3 mL/L and 6 mL/L). Intestinal microbiological parameters, histomorphometric characteristics, and the presence of *Salmonella* Typhimurium in tissues by immunohistochemistry were evaluated. The effects of infection status, HTS concentration, and their interaction were analyzed using two-way ANOVA. HTS administration was associated with reduced counts of total aerobic bacteria, *Escherichia coli* and *Enterobacteriaceae* counts. In experimentally infected broilers, HTS treatment was also associated with reduced *Salmonella* Typhimurium in intestinal tissue and significant changes in intestinal histomorphometric parameters, including increased crypt depth and mitotic activity. The observed responses were influenced by both infection status and the HTS nominal concentration. Because of the limited number of independent experimental units and the absence of individual HTS intake measurements, these findings should be considered exploratory preliminary findings, and further studies with larger animal groups under commercial production conditions are required.

## 1. Introduction

Antimicrobial resistance (AMR) is one of the most important global public health threats and is associated with approximately 35,000 deaths annually in the European Union due to infections caused by resistant bacteria [1,2,3,4]. Recent data from the World Health Organization Global Antimicrobial Resistance and Use Surveillance System (GLASS) further indicate that resistance to critically important antibiotics continues to increase worldwide, particularly among Gram-negative pathogens, emphasizing the urgent need for effective antimicrobial stewardship and complementary disease-control strategies [5]. Despite surveillance programs and measures promoting prudent antimicrobial use, these pathogens continue to emerge and spread. *Enterobacteriaceae*, such as *Klebsiella pneumoniae*, have been increasingly reported with resistance to carbapenems, and *Escherichia coli* with increased potential resistance to third-generation cephalosporins [1,4,5]. In addition, *Salmonella* spp. is a zoonotic foodborne pathogen responsible for intestinal colonization and a major cause of foodborne infections worldwide [6,7,8]. The continued emergence and dissemination of antimicrobial-resistant enteric pathogens highlight the need to develop complementary strategies for the prevention and control of intestinal infections. Consequently, international organizations increasingly advocate integrated approaches combining prudent antimicrobial use, biosecurity, vaccination, nutritional interventions, and phytogenic products to reduce antimicrobial dependence while maintaining animal health and productivity [3,5].

Phytogenic products have attracted considerable interest in poultry production, because of their reported antimicrobial, antioxidant, anti-inflammatory, and immunomodulatory properties [9,10,11,12,13,14,15,16,17,18,19,20,21,22,23,24]. Previous studies have suggested that these bioactive phytocompounds may contribute to maintaining intestinal health, limiting the impact of enteric pathogens, and supporting complementary strategies aimed at reducing antibiotic use in poultry production [15,16,18,20,21,22,23,24]. In addition, experimental studies have demonstrated that phytogenic products and other natural bioactive feed additives may reduce intestinal colonization by *Salmonella* Typhimurium, and were associated with changes in intestinal morphology, and attenuate infection-associated intestinal lesions in broiler chickens [25,26,27]. These findings support the growing interest in phytogenic products as complementary tools for the management of enteric bacterial infections in poultry.

The phytogenic product evaluated in this study contains a hydroalcoholic extract derived from six medicinal plants (*Origanum vulgare*, *Calendula officinalis*, *Mentha* spp., *Hypericum perforatum*, *Plantago major*, and *Achillea millefolium*), selected based on previously reported antimicrobial, anti-inflammatory, and antioxidant properties for their bioactive molecules [28,29,30,31,32,33,34,35,36,37,38,39]. Recent studies have shown that phytogenic blends and other non-antibiotic feed additives were associated with favorable intestinal morphometric responses in broiler chickens, including under experimental *Salmonella* challenge conditions [40,41,42]. However, studies integrating microbiological, histomorphometric, and immunohistochemical evaluations of multi-component phytogenic formulations administered through drinking water under controlled *Salmonella* Typhimurium challenge conditions remain limited.

Based on these considerations, we hypothesized that administering the product Herba Top Salmonella (HTS) through drinking water would be associated with changes in selected cultivable intestinal bacterial populations, intestinal histomorphometric parameters, and the tissue distribution of *Salmonella* Typhimurium in Ross 308 broiler chickens, both uninfected and experimentally infected. Therefore, two nominal HTS concentrations were evaluated by assessing selected cultivable intestinal bacterial populations and intestinal histomorphometric parameters (villi thickness, tunica muscularis thickness, intestinal crypt depth, mitotic index of the intestinal crypt), and *Salmonella* Typhimurium immunoreactivity in intestinal tissues.

By integrating microbiological, histomorphometric, and immunohistochemical findings, the present study aimed to provide additional experimental evidence regarding the responses associated with administration of a multi-component phytogenic water supplement under controlled *Salmonella* Typhimurium challenge conditions. Considering the exploratory nature of the study and the limited number of independent experimental units, the findings should be interpreted with caution and confirmed in larger studies conducted under commercial production conditions. Rather than investigating molecular mechanisms, the study was designed to assess observable biological responses under controlled experimental conditions. The findings provide preliminary experimental evidence that may support future investigations under commercial production conditions.

## 2. Materials and Methods

### 2.1. Product

Herba Top Salmonella (HTS) is a liquid phytogenic formulation consisting of a hydroalcoholic extract obtained from six species of medicinal plants harvested from the spontaneous flora of Mureș County, Romania: *Origanum vulgare* (oregano), *Calendula officinalis* (marigold), *Mentha* spp. (mint), *Hypericum perforatum* (St. John’s wort), *Plantago major* (plantain) and *Achillea millefolium* (yarrow). The phytochemical composition of HTS was previously characterized by Varga et al. [43]. Spectrophotometric analysis indicated a total flavonoid content of 0.82 mg quercetin equivalents (QE)/mL and total polyphenol content of 2.25 mg gallic acid equivalents (GAE)/mL, as well as the presence of representative bioactive compounds, including apigenin (5.075 µg/mL), kaempferol (4.511 µg/mL), quercetin (14.881 µg/mL), luteolin (10.082 µg/mL), quercitrin (33.364 µg/mL), chlorogenic acid (22.573 µg/mL), and caffeic acid (19.218 µg/mL) [43]. Previous studies have associated several of these phytochemical classes with antimicrobial, antioxidant, and anti-inflammatory activities [28,29,30,31,32,33,34,35,36,37,38,39,40]. However, because HTS is a complex phytogenic formulation, the present study was not designed to attribute the observed biological responses to any individual phytochemical.

These concentrations refer to the amount of HTS added to the drinking water rather than the quantity ingested by individual broilers, as water consumption was not monitored at the individual level. Because HTS was administered collectively within each pen, the pen was considered the experimental unit, and all evaluated outcomes were interpreted in relation to the nominal HTS concentrations evaluated under the experimental conditions.

### 2.2. Animals

The experiment was conducted on sixty one-day-old Ross 308 broiler chickens, purchased from a sanitary-veterinary authorized poultry farm. The epidemiological status of the flock was certified as free of *Salmonella* spp. by the supplier and was independently confirmed before experimental challenge by bacteriological examination. The broilers were randomly allocated into six groups (L1–L6; *n* = 10 birds/group), each consisting of two pens (5 birds/pen). Sex was not determined at allocation because chicks were distributed immediately upon arrival. The experimental design included three *Salmonella* Typhimurium-challenged groups: L1 (untreated/positive control); L2 (received HTS in a nominal concentration of 3 mL/L water); L3 (received HTS in a nominal concentration of 6 mL/L water) and three uninfected groups: L4 (received HTS in a nominal concentration of 3 mL/L), L5 (received HTS in a nominal concentration of 6 mL/L water), L6 (untreated/negative control). HTS was administered collectively through drinking water at the pen level, as the experimental unit.

All broiler pens were located within the same animal facility, under identical environmental conditions. Experimentally infected and uninfected groups were housed in physically separated areas. Animal handling and sample collection were always performed starting with the uninfected groups and ending with the infected ones. Disposable protective equipment and consumables were used, and work surfaces and equipment were disinfected between groups, according to the institutional biosafety procedures.

Throughout the experiment, the chickens were monitored clinically daily. Humane endpoints included severe depression, inability to access feed or water, persistent recumbency, severe respiratory distress, or any condition considered incompatible with animal welfare. No animals reached these predefined humane endpoints during the study. No animals or samples collected from animals were excluded from the experiment, all of which were included in the final statistical analyses. Blinding was not applied during sample collection, laboratory processing, or immunohistochemical evaluation because these procedures were performed by the investigators responsible for the experimental analysis. Standardized laboratory protocols and predefined evaluation criteria were applied consistently to all samples to reduce potential observer bias. Details regarding blinding during histopathological evaluation are provided in Section 2.5.

### 2.3. Experimental Protocol

The experiment was conducted in the Biobase of the “Ion Ionescu de la Brad” Iași University of Life Sciences. Experimental infection was induced using *Salmonella enterica* subsp. *enterica* serovar Typhimurium ATCC 14028. The inoculum was prepared from a fresh culture obtained on Mueller-Hinton agar (Oxoid Ltd., Basingstoke, UK), following incubation for 24 h at 37 °C. The bacterial suspension was prepared in sterile saline solution (B. Braun Pharmaceuticals, Melsungen, Germany) at a density of 0.5 McFarland standard, corresponding to a concentration of approximately 1.5 × 10^8^ CFU/mL.

**Figure 1:**
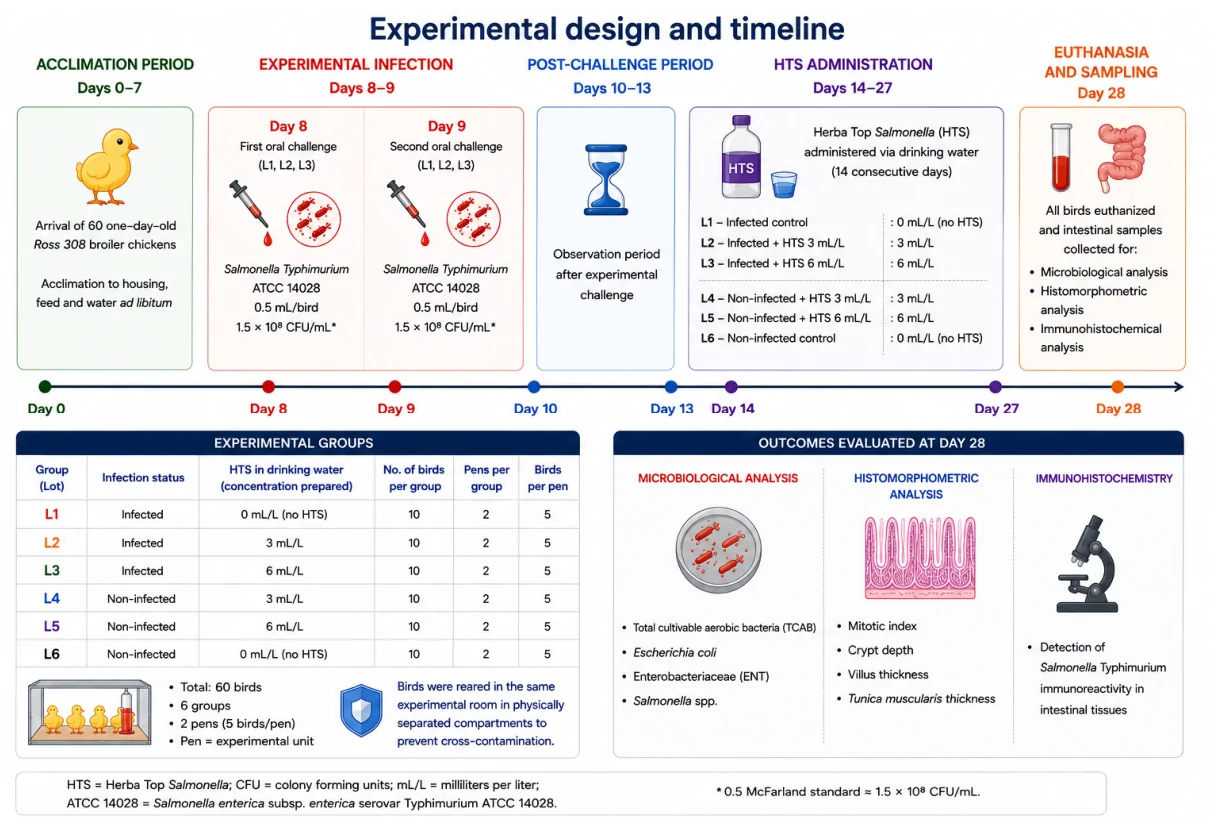
Experimental design and study timeline. Sixty one-day-old Ross 308 broiler chickens were randomly allocated into six experimental groups. Following a 7-day acclimatization period (Days 0–7), birds from groups L1–L3 were orally challenged with *Salmonella enterica* subsp. *enterica* serovar *Typhimurium* ATCC 14028 on days 8 and 9. Herba Top Salmonella (HTS) was administered via drinking water from days 14 to 27 at nominal concentrations of 3 or 6 mL/L On day 28, all birds were euthanized. Cecal contents were collected for microbiological analysis, whereas duodenal samples were collected for histomorphometric and immunohistochemical evaluation.

After seven-day acclimatization period, all broilers from groups L1, L2 and L3 were inoculated orally with 0.5 mL of the bacterial suspension. The procedure was repeated after 24 h, according to the protocol described previously [44]. Five days after the experimental infection, HTS was administered via drinking water for 14 consecutive days, using the two nominal concentrations evaluated in the study (3 mL/L and 6 mL/L) to both infected (L2, L3) and uninfected chickens (L4, L5) (Figure 1). HTS administration was initiated five days after the experimental challenge, in order to evaluate the biological responses associated with therapeutic administration after the infection had been established, rather than as a prophylactic intervention. For each pen, 1 L of freshly prepared HTS solution at the assigned concentration was provided daily. This volume was selected to ensure complete consumption of the phytogenic preparation and consistent administration of the assigned nominal concentration. Complete consumption of the HTS solution was verified daily, after which birds were provided unrestricted access to fresh drinking water (*ad libitum*) for the remainder of the day. Therefore, the HTS solution represented the vehicle for product administration and not the total daily water allowance. Because HTS was administered collectively through drinking water, individual HTS intake could not be determined; therefore, all study outcomes were interpreted in relation to the nominal HTS concentrations prepared in the drinking water rather than to individual ingested doses.

Throughout the experiment, all groups were maintained under identical environmental conditions. Ambient temperature was maintained at 32–34 °C during the first days of life and gradually reduced to 21–24 °C at the end of the experiment. Relative humidity was maintained between 50 and 70%, and the lighting schedule consisted of 24 h of light (natural and artificial) for the first five days, followed by a regime of 18 h of light and 6 h of darkness. Feed and water were provided *ad libitum* throughout the study. No mortality was recorded in any of the experimental groups. At the end of the experiment, all broilers were deeply sedated by intramuscular administration of a ketamine–xylazine combination, followed by decapitation, in accordance with institutional ethical and animal welfare guidelines. All experimental procedures were approved by the Ethics Committee of the Faculty of Veterinary Medicine, “Ion Ionescu de la Brad” Iași University of Life Sciences (Decision No. 1114/24.09.2024).

### 2.4. Bacteriological Examination

At the end of the experiment, following euthanasia, 1 g of cecal content was collected from each broiler. The samples were used to determine the total number of cultivable aerobic bacteria (TCAB), the total number of *Escherichia coli* (*E. coli*), the total number of *Enterobacteriaceae* (ENT) and the presence of *Salmonella* spp. Before the experimental challenge, representative fecal samples were collected and examined for the presence of *Salmonella* spp. using the same qualitative detection protocol described below. All samples tested negative.

For quantitative evaluations, cecal content samples were homogenized in sterile saline, and serial decimal dilutions were prepared. For the enumeration of TCAB, dilutions up to 10^−8^ were prepared, whereas dilutions up to 10^−4^ were sufficient for *E. coli* and *Enterobacteriaceae*. Only plates containing 30–300 colonies were considered suitable for bacterial enumeration. 1 mL of each selected dilution was distributed in sterile Petri dishes and, depending on the parameter evaluated, following culture media, molten and cooled to 45 °C, were used: Mueller–Hinton agar (Bio-Rad Laboratories, Inc., Hercules, CA, USA, cat. no. 64884) for TCAB, RAPID’E.coli 2 Agar (Bio-Rad Laboratories, Inc., Hercules, CA, USA, cat. no. 3555299) for *Escherichia coli*, and RAPID’Enterobacteriaceae Agar (Bio-Rad Laboratories, Inc., Hercules, CA, USA, cat. no. 3554012) for *Enterobacteriaceae* (ENT). After solidification, the plates were incubated at 37 °C for 24 h. Bacterial counts were calculated by multiplying the number of colonies recorded on countable plates by the reciprocal of the corresponding dilution, taking into account the inoculated volume, and were expressed as CFU/g of cecal content. The theoretical detection limit of the enumeration method was 10 CFU/g, based on the pour-plating of 1 mL from the initial 10^−1^ dilution. All microbiological determinations were performed in duplicate. For each broiler, the arithmetic mean of the duplicate determinations was calculated and used as the individual microbiological value. Quantitative results were subsequently log_10_-transformed before statistical analysis.

Detection and confirmation of *Salmonella* spp. was performed according to the standard SR EN ISO 6579-1:2017/Amd1:2020 [45]. For pre-enrichment, samples were mixed with Buffered Peptone Water (BPW) at 37 °C for 18 ± 2 h, followed by selective enrichment in Rappaport–Vassiliadis broth (RVS, Oxoid Ltd., Basingstoke, UK) and Müller–Kauffmann tetrathionate-novobiocin broth (MKTTn, Oxoid Ltd., UK). Isolation was performed on selective XLD medium (Xylose Lysine Deoxycholate, Oxoid Ltd., UK), and suspect colonies were confirmed by MALDI-TOF MS mass spectrometry using the Biotyper^®^ Siri-us One system (Bruker Daltonics GmbH & Co. KG, Bremen, Germany). Because confirmation was performed by MALDI-TOF MS, which identifies isolates at the species level, serological confirmation of the Typhimurium serovar was not performed. This should be considered a limitation of the study.

### 2.5. Histopathological Examination

Immediately after euthanasia, from all broilers included in the study, duodenal samples were collected for histopathological and histomorphometric evaluation, whereas distal ileum samples were collected for immunohistochemical examination. The samples were washed in phosphate-buffered saline (PBS), fixed for 24 h in Bouin’s solution, dehydrated in ethanol, cleared in butanol, and embedded in paraffin wax. Histological sections of 4 µm thickness were stained with hematoxylin–eosin (HE) and examined using a Leica microscope (eica Microsystems GmbH, Wetzlar, Germany) equipped with camera and LAS V4.13 software.

For histomorphometric evaluation, three histological sections were analyzed for each sample, and five microscopic fields were evaluated for each section (15 microscopic fields per broiler). Within each microscopic field, all intact and adequately oriented villi and crypts that met the predefined inclusion criteria were measured. The number of evaluated structures therefore varied among microscopic fields according to tissue preservation and orientation, with a minimum of three villi and three crypts assessed per microscopic field, resulting in at least 45 villi and 45 crypts evaluated per bird. Villi and crypts showing sectioning artifacts, folding, structural disruption, or poor orientation were excluded from the analysis. Villus height was not evaluated because the considerable length and marked variability in villus orientation across tissue sections limited reproducible measurement of this parameter. Many villi were sectioned obliquely or tangentially, preventing consistent identification of the villus tip and the crypt-villus junction. Therefore, villus thickness was selected as an alternative morphometric parameter, measured at the villus base as the distance between two adjacent crypt openings, according to the methodology described by Awad et al. [46]. This parameter could be measured more consistently on well-oriented sections and was considered to provide complementary information to crypt depth and mitotic index.

The evaluated histomorphometric parameters included intestinal villi thickness, crypt depth, and tunica muscularis thickness. The mitotic index was determined by calculating the number of cells in mitosis (cells in prophase, metaphase, anaphase, and telophase) reported per intestinal crypt [47], expressed as the ratio between the number of mitotic figures and the number of intestinal crypts assessed. Villi thickness was measured at the base of the villi, as the distance between two adjacent crypt openings. Crypts were defined as invaginations of the epithelium located between two adjacent villi and their depth was determined from the bottom of the crypts to the base of the villi [46]. Tunica muscularis thickness was assessed on cross sections of the intestinal wall.

For the histopathological initial assessment of all tissue sections, no blinding was used. The morphometric measurements were obtained using predefined, standardized criteria applied uniformly to all samples, which was intended to reduce potential observer bias. To increase the reliability of the evaluation, all histological sections from all experimental groups were subsequently reviewed by a second investigator who was blinded to the experimental group assignment. This independent review was performed to reevaluate independently the histopathological interpretation, and no discrepancies affecting the final interpretation were identified.

Representative photomicrographs were selected from the complete set of histological sections examined during the study to illustrate the characteristic findings observed in each experimental group. Image selection was not performed under blinded conditions. Images were acquired at total magnifications of ×100 and ×1000, corresponding to scale bars of 200 µm and 20 µm, respectively.

The presence and tissue localization of *Salmonella* Typhimurium antigen were evaluated in distal ileum sections by immunohistochemistry (IHC), used as a qualitative method. No quantitative scoring of immunolabeling was performed. Antigen retrieval was performed by microwave heating for 10 min at 95 °C in 10 mM/L citrate buffer (pH 6.0), followed by cooling for 20 min and washing twice in PBS for 5 min each. Endogenous peroxidase activity was blocked with 3% hydrogen peroxide, followed by PBS rinsing. The sections were incubated overnight at 4 °C in a humid chamber with the primary antibody (rabbit polyclonal *Salmonella* antibody—PA1-7244, Thermo Fisher Scientific, Waltham, MA, USA), diluted 1:500. The following day, slides were washed three times in PBS (5 min each) and incubated with the appropriate labeled secondary antibody (goat anti-rabbit IgG, Abcam plc, Cambridge, UK), diluted 1:500. Immunoreactivity was detected using 3,3′-diaminobenzidine (DAB substrate Abcam, plc, Cambridge, UK), followed by haematoxylin counterstaining. A no-primary antibody control was not included because the objective of the IHC analysis was to provide qualitative confirmation of tissue localization using biological positive and negative controls. This should be considered when interpreting the immunohistochemical findings. Intestinal tissues from experimentally infected birds served as positive biological controls, whereas tissues from non-infected birds served as negative biological controls. All controls were processed simultaneously with the test samples under identical conditions. The immunohistochemical evaluation was performed by a single investigator using predefined evaluation criteria.

### 2.6. Statistical Analysis

The distribution of the data was assessed using the Shapiro–Wilk test, and the homogeneity of variances was evaluated using Levene’s test. The biological parameters (microbiological and histomorphological) were obtained from individual broilers. Because HTS was administered collectively through drinking water at the pen level, the pen was considered the experimental unit. Therefore, individual measurements obtained from birds within the same pen were averaged to generate a single value for each pen before statistical analysis. The importance of correctly identifying the experimental unit and avoiding pseudoreplication is emphasized in the ARRIVE 2.0 guidelines [48] and in methodological publications on experimental design [49]. Accordingly, no attempt was made to compensate for the limited number of experimental units by treating individual birds as independent observations. Each experimental group consisted of two pens; therefore, statistical analyses were performed using the two independent pen means obtained for each group. Quantitative microbiological results were logarithmically transformed (log_10_ CFU/g) before calculating the arithmetic mean and analysis. The results are presented as mean per pen ± standard deviation (SD). The effects of infection status (infected vs. uninfected) and HTS concentrations (0, 3, and 6 mL/L) were evaluated by two-way ANOVA, including main effects and factor interactions. For each factor and interaction, F values, significance level (*P*), and effect size (partial eta squared, η^2^p) were reported. When a statistically significant interaction was detected, pairwise comparisons were performed using Tukey’s honestly significant difference (HSD) test. Statistical significance was established at *p* < 0.05. Only statistically significant pairwise comparisons are indicated in the figures. For Tukey HSD post hoc comparisons, adjusted *p*-values together with the corresponding 95% confidence intervals are presented in the Supplementary Materials.

## 3. Results

### 3.1. Bacteriological Examination Results

**Figure 2:**
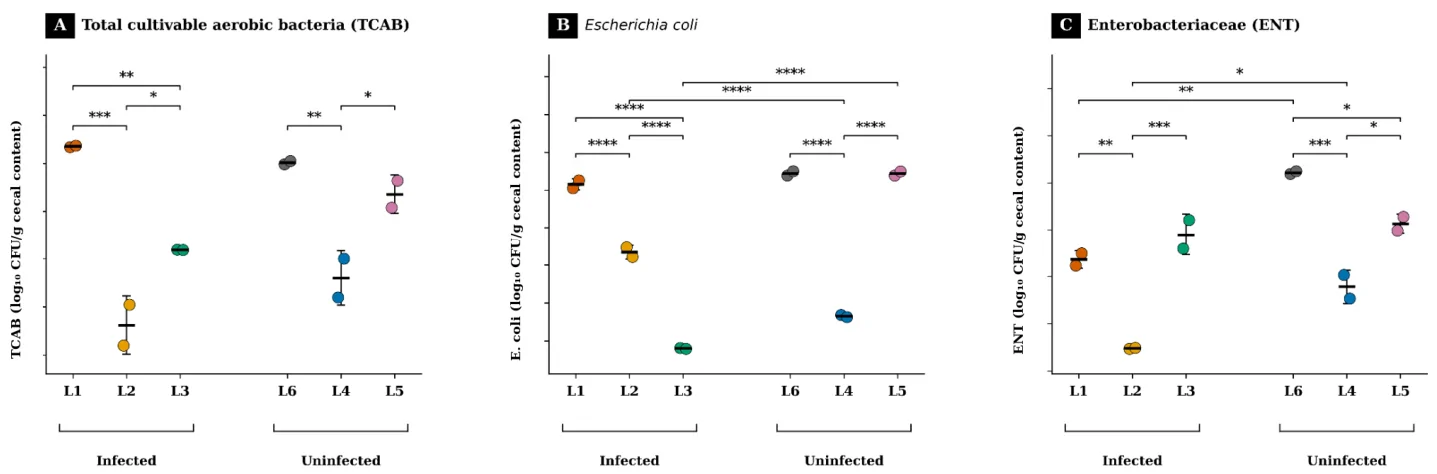
Quantitative cultivable bacterial populations (log_10_ CFU/g cecal content) in Ross 308 broiler experimental groups (L1–L6). Horizontal brackets indicate statistically significant pairwise comparisons identified by Tukey’s honestly significant difference (HSD) test. For TCAB (panel **A**), pairwise comparisons corresponding to the significant main effect of HTS concentration. For *E. coli* (panel **B**) and *Enterobacteriaceae* (panel **C**), pairwise comparisons are shown only when a significant interaction between infection status and HTS concentration was detected. Significance levels are indicated as * *p* < 0.05, ** *p* < 0.01, *** *p* < 0.001, **** *p* < 0.0001. Data are presented as mean ± SD of the two independent pen means per experimental group.

Microbiological evaluation of cecal content revealed significant differences among the experimental groups, regarding total cultivable aerobic bacteria (TCAB), *Escherichia coli*, and *Enterobacteriaceae* (ENT) (Table 1 and Table S1, Supplementary Materials, Figure 2).

Two-way ANOVA demonstrated significant effects of infection status and HTS concentration on all evaluated microbiological parameters (Table 1). For TCAB, significant main effects of infection status (F(1,6) = 7.79; *p* = 0.032) and HTS concentration (F(2,6) = 67.5; *p* = 0.0001) were observed, whereas their interaction was not statistically significant (F(2,6) = 4.83; *p* = 0.056). For *E. coli*, significant effects of infection status (F(1,6) = 304.29; *p* < 0.0001), HTS concentration (F(2,6) = 752.07, *p* < 0.0001), and their interaction (F(2,6) = 909.69; *p* < 0.0001) were detected. Similarly, *Enterobacteriaceae* counts were significantly affected by infection status (F(1,6) = 58.35; *p* = 0.0003), HTS concentration (F(2,6) = 84.07; *p* < 0.0001), and the interaction between the two factors (F(2,6) = 10.35; *p* = 0.011).

Because significant interactions were identified for *E. coli* and *Enterobacteriaceae*, post hoc analyses (Tukey HSD) were performed. In infected broilers, administration of HTS at 3 mL/L (L2) and 6 mL/L (L3) was associated with significantly lower *E. coli* counts, compared to the untreated infected group (L1) (L1 vs. L2: *p* < 0.0001; L1 vs. L3: *p* < 0.0001), while the two HTS concentrations also differed significantly from each other (L2 vs. L3: *p* < 0.0001). Among uninfected broilers, *E. coli* counts differed significantly between birds receiving 3 and 6 mL/L (L4 vs. L5, *p* < 0.0001). Significant differences were also observed between infected and uninfected broilers receiving the same HTS concentration, both at 3 mL/L (L2 vs. L4: *p* < 0.0001) and at 6 mL/L (L3 vs. L5: *p* < 0.0001). For *Enterobacteriaceae*, significant differences were detected between L1 and L2 (*p* = 0.003), L4 and L5 (*p* = 0.014), L1 and L6 (*p* = 0.003), and L2 and L4 (*p* = 0.017). Complete Tukey HSD results, including adjusted *p* values and 95% confidence intervals, are presented in Tables S3–S5 (Supplementary Materials).

Percentage and log_10_ reductions were calculated as descriptive indicators to facilitate comparisons between experimental groups (Table S6, Supplementary Materials). In infected broilers, administration of HTS at 3 mL/L was associated with reductions of 57.5% (0.37 log_10_) for TCAB, 64.8% (0.45 log_10_) for *E. coli*, and 35.2% (0.19 log_10_) for *Enterobacteriaceae* compared with the untreated infected control. Administration at 6 mL/L was associated with reductions of 40.5% (0.23 log_10_) for TCAB and 91.9% (1.09 log_10_) for *E. coli*. In uninfected broilers, HTS at 3 mL/L was associated with reductions of 42.4% (0.24 log_10_) for TCAB, 88.8% (0.95 log_10_) for *E. coli*, and 42.9% (0.24 log_10_) for *Enterobacteriaceae*, whereas administration at 6 mL/L resulted in smaller reductions (13.8%, 0.06 log_10_ for TCAB; 22.1%, 0.11 log_10_ for both *E. coli* and *Enterobacteriaceae*). These percentage and logarithmic reductions are presented as descriptive measures and should not be interpreted independently of the statistical analyses.

*Salmonella* spp. was isolated exclusively from the untreated infected group (L1). The identity of the isolates was confirmed by MALDI-TOF mass spectrometry, whereas no *Salmonella* spp. was detected in any HTS-treated or uninfected group.

### 3.2. Histopathological Examination Results

**Figure 3:**
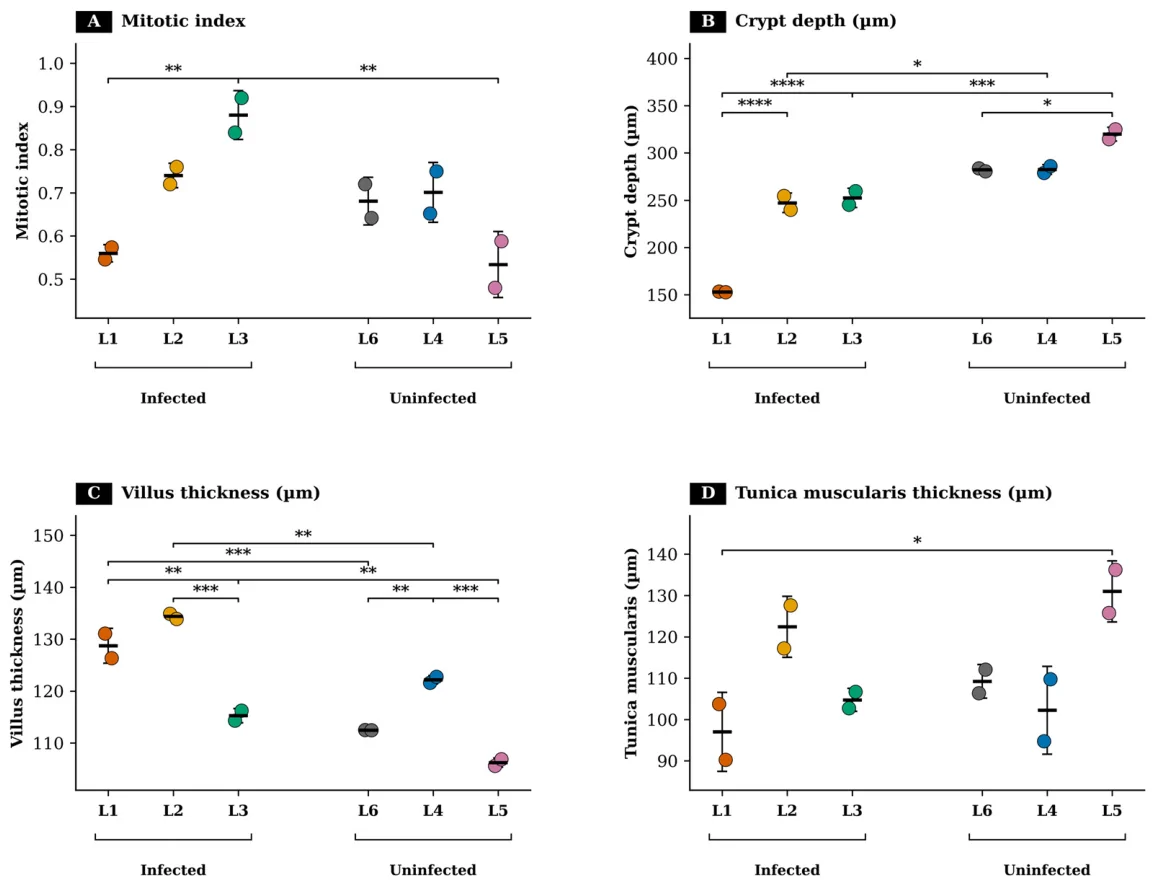
Histomorphometric parameters evaluated in the duodenum of Ross 308 broiler experimental groups (L1–L6). (**A**) Mitotic index; (**B**) Crypt depth; (**C**) Villus thickness; (**D**) Tunica muscularis thickness. Horizontal brackets indicate statistically significant pairwise comparisons identified by Tukey’s honestly significant difference (HSD) post hoc test. Only statistically significant comparisons are shown (* *p* < 0.05; ** *p* < 0.01; *** *p* < 0.001; **** *p* < 0.0001). Data are presented as mean ± SD of pen-level means (two pens per experimental group).

Histomorphometric evaluation revealed significant variations among the experimental groups in mitotic index, villi thickness, tunica muscularis thickness, and intestinal crypt depth. Two-way ANOVA showed significant effects of infection status, nominal HTS concentration, and their interaction on all analyzed parameters (Table S7, Supplementary Materials, Figure 3).

The mitotic index was significantly influenced by infection status (F(1,6) = 7.71; *p* = 0.032) and by the interaction between infection and nominal HTS concentration (F(2,6) = 18.70; *p* = 0.003), whereas the main effect of nominal HTS concentration was not statistically significant (F(2,6) = 3.91; *p* = 0.082). Post hoc analyses (Tukey HSD) showed that, within the infected group, broilers receiving the nominal HTS concentration of 6 mL/L (L3) showed a significantly higher mitotic index compared to the untreated group (L1) (*p* = 0.008). In addition, the mitotic index was significantly higher in L3 than in the corresponding uninfected group receiving the same nominal HTS concentration (L5) (*p* = 0.006). No other statistically significant differences were identified (Table 2 and Table S8, Supplementary Materials).

Intestinal crypt depth was significantly affected by infection status (F(1,6) = 368.92; *p* < 0.0001), nominal HTS concentration (F(2,6) = 101.30; *p* < 0.0001) and the interaction between these factors (F(2,6) = 46.79; *p* = 0.0002). Post hoc analysis (Table S9, Supplementary Materials) showed that, within the infected groups, both nominal HTS concentrations were associated with a significant increase in crypt depth, compared to the untreated group (L1 vs. L2: *p* < 0.0001; L1 vs. L3: *p* < 0.0001). Among the uninfected groups, broilers receiving the nominal HTS concentration of 6 mL/L (L5) showed significantly higher values, compared to those receiving 3 mL/L (L4) (*p* = 0.013). Significant differences were also observed between infected and uninfected broilers receiving the same nominal HTS concentration, both at 3 mL/L (L2 vs. L4: *p* = 0.017) and at 6 mL/L (L3 vs. L5: *p* = 0.0006). In addition, the untreated infected group (L1) showed significantly lower crypt depth than the untreated uninfected control group (L6) (*p* < 0.0001).

Intestinal villi thickness was significantly affected by infection status (F(1,6) = 186.91; *p* < 0.0001), nominal HTS concentration (F(2,6) = 123.24; *p* < 0.0001) and the interaction between these two factors (F(2,6) = 5.20; *p* = 0.049). Post hoc analyses (Table S10, Supplementary Materials) showed that, within the infected groups, broilers receiving the nominal HTS concentration of 6 mL/L (L3) were associated with significantly lower values of villi thickness, compared to the untreated group (L1) (*p* = 0.001). Among the uninfected groups, broilers receiving 6 mL/L (L5) also showed significantly lower values, compared to those receiving 3 mL/L (L4) (*p* = 0.0004). In addition, significant differences were observed between the infected and uninfected groups receiving the nominal HTS concentration of 3 mL/L (L2 vs. L4; *p* = 0.002).

Tunica muscularis thickness was not significantly affected by infection status (F(1,6) = 1.99; *p* = 0.208) or nominal HTS concentration (F(2,6) = 3.95; *p* = 0.081), whereas the interaction between these two factors was statistically significant (F(2,6) = 10.09; *p* = 0.012). Post hoc analyses (Table S11, Supplementary Materials) revealed a significant difference between the untreated infected group (L1) and the uninfected group receiving the nominal HTS concentration of 6 mL/L HTS (L5) (*p* = 0.028). No other statistically significant pairwise differences were identified.

Overall, the most pronounced pairwise differences were observed for intestinal crypt depth, whereas fewer significant differences were identified for mitotic index, villi thickness, and tunica muscularis thickness.

**Figure 4:**
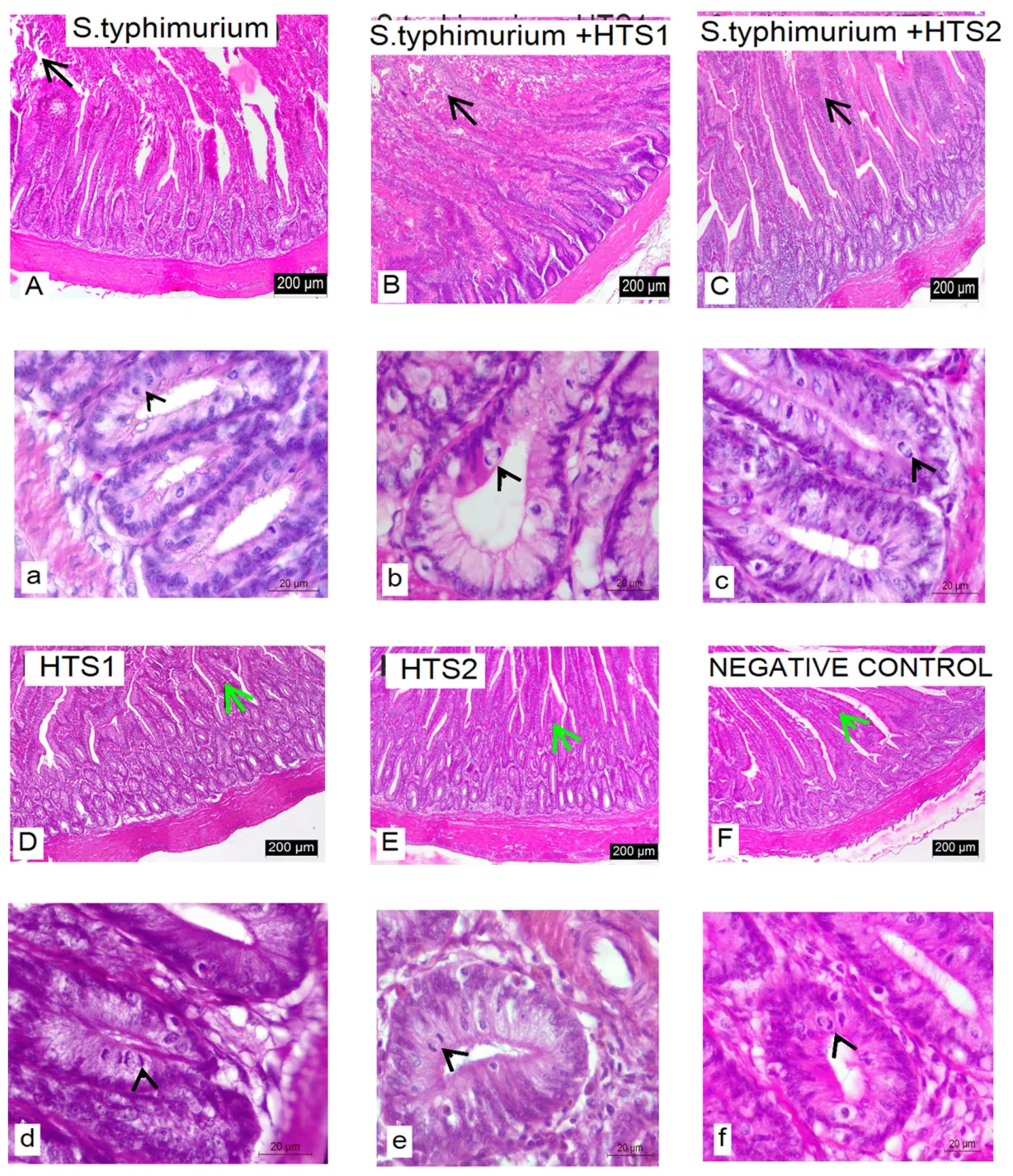
Histopathological evaluation of the duodenum in Ross 308 broiler chickens from the experimental groups. Representative hematoxylin and eosin (H&E)-stained duodenal sections. Upper panels (**A**–**F**) show representative low-magnification views of the duodenal wall (scale bar = 200 μm), whereas lower panels (**a**–**f**) show higher-magnification views of the corresponding areas, highlighting intestinal crypt morphology and mitotic figures (scale bar = 20 μm). (**A**,**a**) *Salmonella* Typhimurium-infected untreated broilers (L1); (**B**,**b**) infected broilers treated with HTS at 3 mL/L (L2); (**C**,**c**) infected broilers treated with HTS at 6 mL/L (L3); (**D**,**d**) uninfected broilers treated with HTS at 3 mL/L (L4); (**E**,**e**) uninfected broilers treated with HTS at 6 mL/L (L5); (**F**,**f**) uninfected untreated control broilers (L6). Black arrows indicate villi with degenerative changes, green arrows indicate villi with preserved normal architecture, and arrowheads indicate mitotic figures within the intestinal crypts.

Histopathological examination of the duodenum revealed structural differences among the experimental groups, according to infection status and HTS administration. In the infected untreated group (L1), the intestinal villi exhibited degenerative changes, characterized by apical exfoliation of the epithelium, subepithelial edema, reduced villi thickness, and villi fusion. Leukocyte infiltrates and vascular congestion were observed in the lamina propria, whereas the intestinal crypts appeared hyperplastic and dilated (Figure 4).

In infected group receiving 3 mL/L (L2), histological alterations appeared less pronounced than those observed in L1, and were characterized by moderate epithelial detachment, subepithelial edema, occasional fused villi, apparently reduced inflammatory leukocyte infiltration within the lamina propria, and moderate crypt hyperplasia. In the infected group receiving 6 mL/L HTS (L3), the intestinal mucosa also exhibited reduced inflammatory changes compared with L1, although deeper crypts and thinner villi were observed in agreement with the histomorphometric measurements. In the uninfected groups receiving HTS (L4 and L5), the overall intestinal architecture was comparable to that observed in the uninfected and untreated control group. Group L4 showed preserved villus architecture with an intact epithelial lining, normal crypt morphology, and no evident inflammatory infiltrates. Group L5 exhibited preserved mucosal organization with deeper crypts, thinner villi, and an intact tunica muscularis. The untreated uninfected control group (L6) showed normal duodenal histological architecture. Villus height and the villus height-to-crypt depth ratio were not evaluated in this study because of the methodological considerations described in Section 2.5. Therefore, the histomorphometric findings should be interpreted within the context of the parameters assessed.

**Figure 5:**
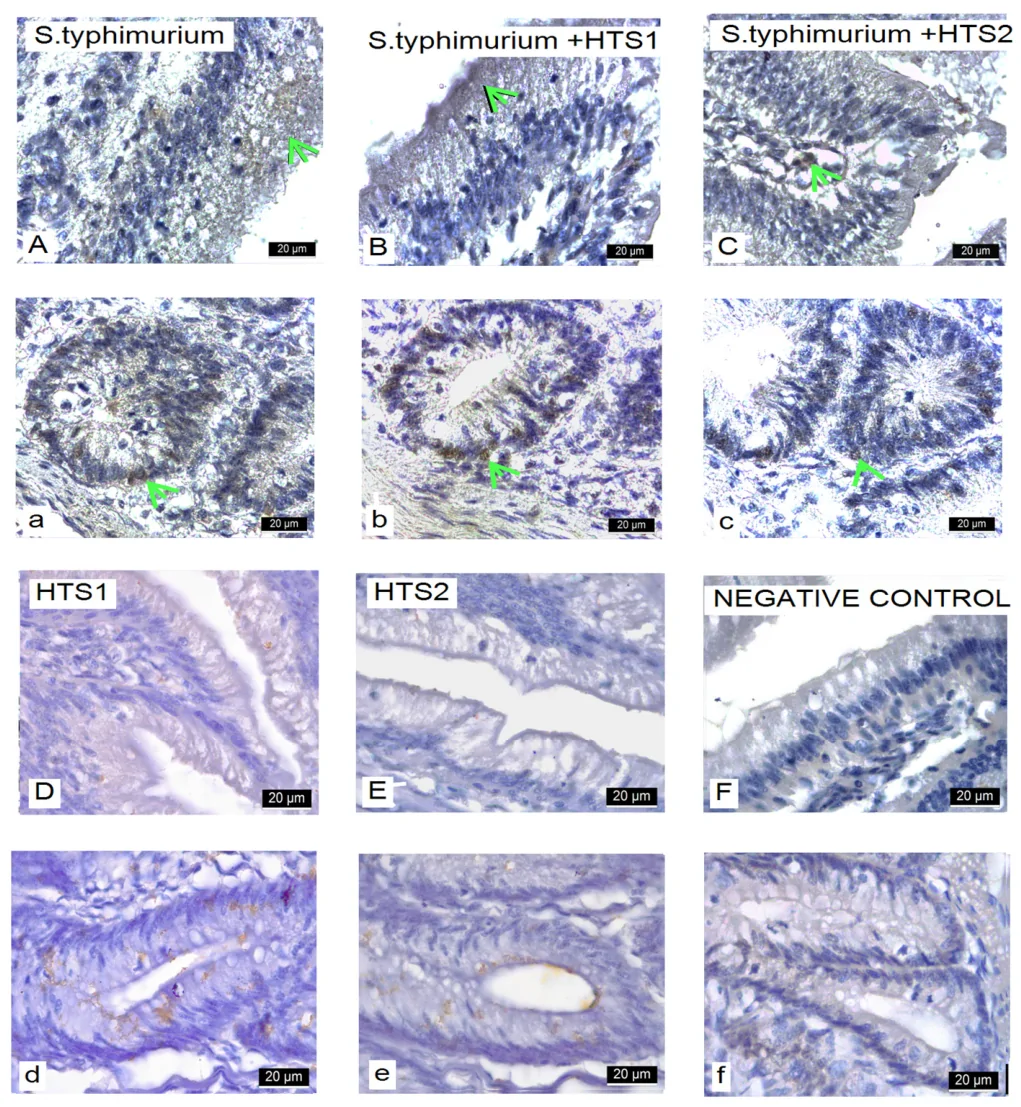
Immunohistochemical detection of *Salmonella* Typhimurium antigen in the distal ileum of Ross 308 broiler chickens. Representative sections immunostained with a rabbit polyclonal anti-*Salmonella* antibody and counterstained with hematoxylin. Upper panels (**A**–**F**) show representative views of the distal ileal mucosa, whereas lower panels (**a**–**f**) show higher-magnification views of the corresponding areas, illustrating immunoreactivity within the intestinal crypts (scale bar = 20 μm). (**A**,**a**) *Salmonella* Typhimurium-infected untreated broilers (L1); (**B**,**b**) infected broilers treated with HTS at 3 mL/L (L2); (**C**,**c**) infected broilers treated with HTS at 6 mL/L (L3); (**D**,**d**) uninfected broilers treated with HTS at 3 mL/L (L4); (**E**,**e**) uninfected broilers treated with HTS at 6 mL/L (L5); (**F**,**f**) uninfected untreated broilers (L6). Green arrows (upper panels) and arrowheads (lower panels) indicate positive immunoreactivity for *Salmonella* Typhimurium antigen. No specific immunoreactivity was observed in the uninfected groups.

Immunohistochemical evaluation demonstrated the presence and tissue localization of *Salmonella* Typhimurium antigen exclusively in the experimentally infected groups (Figure 5). In the untreated infected group (L1), positive immunolabeling was observed in cells located predominantly within the intestinal crypt region, consistent with macrophages and intraepithelial lymphocytes. In the HTS-treated infected groups (L2 and L3), positive immunolabeling was also detected. However, the distribution of immunopositive cells appeared less extensive than that observed in the untreated infected group (L1). No immunopositive cells were detected in the uninfected groups (L4, L5, and L6).

## 4. Discussion

The continuing global increase in antimicrobial resistance, together with progressively stricter regulations governing antimicrobial use in food-producing animals, has intensified the search for complementary strategies capable of supporting intestinal health while reducing dependence on conventional antibiotics. Recent data from the World Health Organization Global Antimicrobial Resistance and Use Surveillance System (GLASS) indicate that resistance to critically important antimicrobials continues to increase worldwide, particularly among Gram-negative pathogens, reinforcing the need for evidence-based interventions that support antimicrobial stewardship within the One Health framework [5,13,15,16]. In this context, phytogenic formulations have attracted considerable interest because they contain multiple bioactive compounds with antimicrobial, antioxidant, anti-inflammatory, and immunomodulatory properties that may act through multiple complementary mechanisms resulting from the combined activity of their bioactive constituents rather than from a single compound [18,19,20,21,22,23,24,25,50]. Nevertheless, despite the growing number of studies investigating phytogenic products in poultry, substantial differences in botanical composition, extraction procedures, administration routes, challenge models, and evaluated outcomes limit direct comparisons among studies and complicate interpretation of their biological effects. Consequently, each phytogenic formulation should be interpreted according to its own botanical composition and experimental conditions rather than by direct extrapolation from studies evaluating different plant-derived products.

The present exploratory study evaluated the association between administration of the phytogenic formulation HTS and intestinal responses in Ross 308 broilers experimentally challenged with *Salmonella enterica* subsp. *enterica* serovar Typhimurium. Rather than focusing on a single endpoint, the study combined conventional bacteriological examination of cecal content with histopathological, histomorphometric, and immunohistochemical evaluation of intestinal tissues, allowing assessment of complementary aspects of the host response to experimental infection and phytogenic supplementation. Under the experimental conditions applied, HTS administration was associated with differences in selected cultivable bacterial populations, histomorphometric parameters, and the qualitative tissue distribution of *Salmonella* antigen. Because the study was designed as an exploratory investigation and was not intended to elucidate molecular mechanisms, the observed findings should be interpreted as biologically relevant associations requiring independent confirmation in larger studies incorporating quantitative microbiological, microbiome, immunological, and molecular approaches.

One of the earliest consequences of intestinal *Salmonella* infection is disruption of the intestinal microbial ecosystem, resulting in altered microbial composition, epithelial dysfunction, and activation of local immune responses. The intestinal microbiota contributes to host protection by competing with invading pathogens for nutrients and ecological niches, producing antimicrobial metabolites, and maintaining epithelial barrier integrity. Consequently, changes in cultivable bacterial populations observed during experimental salmonellosis should not be interpreted solely as alterations in bacterial counts but rather as indicators of broader ecological changes occurring within the intestinal environment [41,44].

In the present study, HTS administration was associated with reductions in cultivable *Escherichia coli*, *Enterobacteriaceae*, and total cultivable aerobic bacteria in cecal content. Because these microbiological evaluations were based on conventional culture techniques, they reflect changes in selected cultivable bacterial populations rather than the overall intestinal microbiota. Nevertheless, when interpreted together with the histopathological, histomorphometric, and immunohistochemical findings, these results suggest that HTS administration was associated with changes in the intestinal environment following experimental *Salmonella* challenge.

These observations are consistent with previous experimental studies demonstrating that phytogenic products may contribute to maintaining intestinal microbial balance during enteric infections. Bescucci et al. [44] showed that experimental infection with *Salmonella enterica* serovar *Typhimurium* induced marked alterations in the enteric microbiota together with changes in host immune responses and metabolic pathways, emphasizing that intestinal salmonellosis is not merely a localized bacterial infection but a complex disturbance of the intestinal ecosystem. Although the present study did not include microbiome sequencing or metabolomic analyses, the combined microbiological and tissue findings are consistent with the concept that phytogenic supplementation may contribute to changes the intestinal milieu during bacterial challenge.

The growing interest in phytogenic formulations reflects international efforts to reduce antimicrobial use in food-producing animals while preserving animal health and productivity [13,14,15,16]. Unlike conventional antibiotics, which primarily exert direct bactericidal or bacteriostatic effects, phytogenic products are believed to influence intestinal health through multiple complementary mechanisms, including interference with bacterial adhesion and quorum sensing, disruption of microbial membranes, antioxidant activity, modulation of inflammatory pathways, and interactions with the intestinal microbiota and host immune system [18,23,24,50]. Consequently, the biological activity of phytogenic preparations is generally attributed to the synergistic action of numerous bioactive compounds rather than to a single active constituent.

HTS contains several flavonoid fractions, including quercetin, quercitrin, luteolin, apigenin, and kaempferol, together with phenolic acids such as chlorogenic and caffeic acids. These classes of phytochemicals have individually been associated with antimicrobial, antioxidant, anti-inflammatory, and epithelial protective properties in poultry [51,52,53,54,55,56,57,58]. However, because HTS was evaluated as an entire phytogenic formulation, the present findings should be interpreted as reflecting the combined biological activity of the formulation rather than the effect of any individual constituent.

Likewise, chlorogenic acid has been reported to alleviate intestinal injury, improve epithelial barrier function, and promote a more favorable intestinal environment in experimentally challenged broilers [59,60,61]. In addition, *Origanum vulgare*, one of the principal botanical components of HTS, contains phenolic monoterpenes including carvacrol and thymol, compounds that have repeatedly demonstrated antimicrobial activity through disruption of bacterial membrane integrity and interference with microbial metabolism [28]. It is important to emphasize that HTS should be regarded as a complex phytogenic formulation rather than as a source of isolated phytochemicals. Although several constituents identified by HPLC have individually been associated with antimicrobial, antioxidant, and immunomodulatory activities, the present experimental design does not allow attribution of the observed biological responses to any single compound. Instead, the measured effects most likely reflect the combined biological activity of multiple flavonoid fractions, phenolic acids, terpenoids, and other plant-derived constituents present in the formulation. This interpretation is consistent with the current understanding of phytogenic feed additives, whose biological activity is generally considered to arise from multiple complementary mechanisms rather than from the action of an individual phytochemical [23,24,50].

Direct quantitative comparison with previously published studies should nevertheless be interpreted cautiously. Considerable methodological differences exist among experimental models, including the botanical composition of phytogenic formulations, extraction procedures, administration through feed or drinking water, duration of supplementation, bacterial challenge protocols, sampled intestinal segments, and microbiological methodologies. Moreover, the present investigation assessed only cultivable bacterial populations and qualitative bacteriological detection of Salmonella spp. and qualitative immunohistochemical detection of *Salmonella* antigen, whereas several recent studies have combined microbiological analyses with molecular characterization of the intestinal microbiota. Consequently, additional investigations integrating culture-dependent techniques with next-generation sequencing, quantitative determination of *Salmonella* colonization, and host immune profiling would be valuable to better define the mechanisms underlying the biological responses associated with HTS administration.

Experimental infection with *Salmonella enterica* serovar Typhimurium is known to induce a complex sequence of pathological events involving epithelial injury, inflammatory cell recruitment, crypt hyperplasia, and disruption of intestinal barrier function. The severity of these alterations depends on multiple factors, including the infective dose, duration of infection, intestinal segment examined, host genetics, and the local immune response. Histopathological evaluation therefore provides important complementary information to bacteriological examination because it reflects the structural consequences of bacterial colonization and the host response to infection rather than bacterial presence alone.

In the present study, the untreated infected broilers exhibited characteristic lesions of experimental salmonellosis, including epithelial exfoliation, villus fusion, subepithelial edema, vascular congestion, inflammatory cell infiltration within the lamina propria, and crypt hyperplasia. These observations are consistent with previous descriptions of intestinal lesions induced by *Salmonella* in broiler chickens and support the effectiveness of the experimental challenge. Similar structural alterations have been reported in experimental infection models in which *Salmonella* compromises epithelial integrity, promotes inflammatory infiltration, and stimulates epithelial regeneration through activation of crypt cell proliferation [44,62].

Interestingly, although histological alterations were still evident in HTS-treated infected broilers, they appeared less extensive than those observed in the untreated infected controls. Rather than indicating complete protection against intestinal injury, these observations suggest that HTS administration was associated with attenuation of some morphological changes accompanying experimental infection. Because no standardized semiquantitative histological scoring system was incorporated into the experimental protocol, these findings should be regarded as descriptive and should not be interpreted as quantitative evidence of improved intestinal integrity. Nevertheless, the qualitative observations were consistent with the morphometric measurements and therefore provide complementary evidence supporting the overall tissue response observed in treated birds.

The biological plausibility of these observations is supported by previous experimental studies demonstrating that phytogenic compounds may contribute to preservation of intestinal morphology under conditions of infectious or inflammatory stress. Chlorogenic acid has been reported to reduce intestinal injury, oxidative stress, and inflammatory responses in broilers challenged with *Clostridium perfringens*, while simultaneously improving epithelial barrier function [59]. Similar beneficial effects on intestinal morphology have been reported following dietary supplementation with quercetin and other flavonoids through attenuation of oxidative damage and modulation of inflammatory pathways [51,52,58]. Furthermore, recent reviews indicate that phytogenic formulations containing multiple polyphenolic compounds may contribute to maintenance of epithelial homeostasis by limiting excessive inflammatory responses and preserving mucosal architecture under enteric challenge conditions [23,24,50]. Because HTS contains several medicinal plants providing numerous bioactive compounds, the present observations most likely reflect the combined activity of multiple phytochemicals rather than the action of a single constituent.

Immunohistochemistry provided additional information regarding the tissue distribution of *Salmonella* antigen. Immunopositive cells were detected exclusively in experimentally infected birds, predominantly within mononuclear cells located around the intestinal crypts, morphologically consistent with macrophages and intraepithelial lymphocytes, whereas no immunolabeling was observed in tissues from uninfected broilers. This localization agrees with the known pathogenesis of *Salmonella*, which invades the intestinal epithelium through specialized epithelial cells and is subsequently internalized by professional phagocytic cells within the intestinal mucosa [44]. Detection of immunopositive mononuclear cells therefore reflects the persistence of bacterial antigen within host immune cells rather than the simple presence of extracellular bacteria.

An apparent discrepancy was observed between bacteriological culture and immunohistochemical findings, as positive bacterial cultures were obtained only from the untreated infected group, whereas immunolabeling was detected in all infected groups, although less extensively in HTS-treated birds. This observation is biologically plausible because the two methods evaluate different biological targets. Conventional bacteriology detects viable organisms capable of replication under culture conditions, whereas immunohistochemistry identifies bacterial antigen that may persist within phagocytic cells after bacterial inactivation or clearance. Consequently, persistence of immunoreactivity should not be interpreted as evidence of ongoing viable infection but rather as an indicator of previous bacterial exposure and antigen retention within intestinal tissues.

Overall, the histopathological and immunohistochemical findings complement the bacteriological results by demonstrating that HTS administration was associated with qualitative differences in intestinal tissue responses following experimental *Salmonella* challenge. However, because quantitative immunohistochemical scoring and standardized histological lesion grading were not included in the experimental design, these observations should be interpreted as supportive rather than definitive evidence and warrant confirmation in future investigations using quantitative image analysis and validated histopathological scoring systems.

Intestinal epithelial integrity is maintained through a continuous process of epithelial renewal in which stem cells located within the intestinal crypts proliferate, differentiate, and migrate toward the villus tip, replacing mature epithelial cells that are continuously lost through physiological desquamation. During enteric infections, this dynamic equilibrium is profoundly altered because epithelial injury accelerates cell loss while simultaneously stimulating compensatory crypt proliferation to restore mucosal integrity [62]. Consequently, histomorphometric parameters such as mitotic index, crypt depth, and villus morphology should be interpreted collectively as indicators of epithelial remodeling rather than as isolated markers of intestinal health.

In the present study, experimental *Salmonella* infection and HTS administration were associated with significant changes in epithelial proliferative activity and intestinal architecture. The increased mitotic index observed in HTS-treated infected broilers suggests enhanced epithelial cell proliferation within the intestinal crypts. Increased crypt cell proliferation is generally regarded as a physiological response to epithelial injury because intestinal stem cells become activated to replace damaged enterocytes and restore mucosal continuity [62]. Therefore, a higher mitotic index should not automatically be interpreted as evidence of improved intestinal function but rather as an indicator of increased epithelial turnover occurring during tissue adaptation and repair.

These observations are consistent with the known pathophysiology of *Salmonella* infection. Xie et al. [62] demonstrated that *Salmonella* infection stimulates crypt hyperplasia in chickens through activation of the Wnt/β-catenin signaling pathway, resulting in increased epithelial proliferation within the intestinal crypts. Consequently, the higher proliferative activity observed in HTS-treated birds may reflect modulation of epithelial renewal during recovery rather than direct stimulation of epithelial growth.

Crypt depth showed a similar pattern of response. Enlargement of intestinal crypts is frequently interpreted as evidence of increased epithelial proliferative activity because proliferating progenitor cells are concentrated within this compartment. However, deeper crypts are not inherently beneficial or detrimental. Depending on the biological context, they may indicate active tissue regeneration, compensatory epithelial renewal following injury, or persistent inflammatory stimulation [41,62]. Interpretation therefore requires integration with the accompanying histopathological findings and other morphometric parameters. In the present study, the increased crypt depth observed in treated broilers occurred together with qualitative histological evidence of less extensive epithelial alterations than those observed in untreated infected birds, suggesting that increased proliferative activity may have represented part of the adaptive mucosal response following experimental challenge rather than isolated pathological hyperplasia.

Unlike crypt depth, villus thickness is not among the conventional histomorphometric parameters most frequently reported in poultry studies, where villus height and the villus height-to-crypt depth ratio are generally preferred [46]. In the present investigation, villus thickness was selected because the considerable length and variable orientation of intestinal villi limited reproducible measurement of villus height across all tissue sections. Measurements were therefore restricted to well-oriented villi in which the distance between adjacent crypt openings could be determined consistently. Although measurements of villus thickness have occasionally been reported in poultry histomorphometric studies, they remain less frequently used than villus height and should therefore be interpreted together with complementary histopathological findings.

Only limited changes were observed in tunica muscularis thickness. The intestinal muscular layer plays an important role in peristalsis, intestinal transit, and coordination of local inflammatory responses, yet structural remodeling of this layer generally occurs later than epithelial alterations during enteric disease. Accordingly, the isolated differences observed in the present study should be interpreted with caution because no consistent pattern was evident across experimental groups, suggesting that HTS administration exerted a more pronounced influence on epithelial rather than muscular components of the intestinal wall.

The overall interpretation of the histomorphometric findings should therefore consider the coordinated behavior of all evaluated parameters rather than individual measurements. Histopathological examination demonstrated epithelial injury in untreated infected broilers, whereas morphometric evaluation revealed concurrent changes in epithelial proliferation and crypt architecture. Together with the less extensive distribution of *Salmonella* antigen observed by immunohistochemistry in treated birds, these findings suggest that HTS administration was associated with modification of intestinal epithelial responses following experimental *Salmonella* challenge. Nevertheless, because conventional indices such as villus height and the villus height-to-crypt depth ratio were not evaluated, and no standardized histological lesion scoring system was incorporated into the study design, these observations should be interpreted as exploratory and require confirmation using comprehensive morphometric and functional assessments in future investigations.

The present study possesses several strengths that should be considered when interpreting the findings. Unlike many experimental investigations focusing on a single outcome, the biological responses associated with HTS administration were evaluated using complementary bacteriological, histopathological, histomorphometric, and immunohistochemical approaches. This integrative experimental design allowed interpretation of bacterial findings together with tissue alterations and epithelial remodeling, providing a broader overview of host responses during experimental *Salmonella* infection than would have been achieved using any individual methodology alone. Furthermore, the study was conducted under controlled experimental conditions using a well-characterized *Salmonella* Typhimurium challenge strain and predefined evaluation criteria, reducing methodological variability among experimental groups.

Nevertheless, several limitations should also be acknowledged. First, the study was designed as an exploratory investigation and included only two independent experimental units per treatment group, consistent with the experimental design adopted to minimize animal use in accordance with the principles of the 3Rs and the ARRIVE 2.0 recommendations [48]. Consequently, although the statistical analyses were performed using the pen as the experimental unit, confirmation in larger studies with a greater number of biological replicates is warranted. Second, microbiological evaluation relied primarily on conventional culture techniques and endpoint qualitative detection of *Salmonella* spp. Consequently, neither comprehensive characterization of the intestinal microbiota nor quantitative assessment of *Salmonella* colonization was possible. Likewise, histopathological evaluation was descriptive and was not supported by a predefined semiquantitative lesion scoring system, while immunohistochemistry was intentionally used as a qualitative method to assess tissue localization of *Salmonella* antigen rather than quantitative bacterial burden. Finally, because HTS was evaluated as a multicomponent phytogenic formulation, the contribution of individual bioactive compounds to the observed biological responses cannot be determined.

These limitations do not diminish the value of the present findings but rather define the scope within which they should be interpreted. Collectively, the bacteriological, histopathological, histomorphometric, and immunohistochemical observations are consistent with HTS administration being associated with measurable changes in intestinal responses following experimental *Salmonella* challenge. Future investigations integrating quantitative microbiological techniques, high-throughput microbiome sequencing, standardized histopathological scoring, digital image analysis, molecular characterization of host immune responses, and direct comparisons with conventional antimicrobial strategies will be important to further clarify the mechanisms underlying these associations and to establish the practical relevance of phytogenic formulations in poultry health management.

Taken together, the present findings support further investigation of multicomponent phytogenic formulations as complementary approaches for improving intestinal health in poultry while contributing to strategies aimed at reducing antimicrobial use.

## 5. Conclusions

Under the experimental conditions of the present study, administration of the phytogenic formulation HTS was associated with changes in selected cultivable intestinal bacterial populations, intestinal histopathology, epithelial proliferative activity, and tissue distribution of *Salmonella* antigen in experimentally challenged Ross 308 broilers. The combined bacteriological, histopathological, histomorphometric, and immunohistochemical findings suggest that HTS administration was associated with measurable changes in intestinal responses during experimental *Salmonella Typhimurium* infection.

Because this exploratory study was not designed to investigate molecular mechanisms or establish causal relationships, the observed associations should be interpreted with appropriate caution. Nevertheless, the integrated evaluation of intestinal microbiology and tissue responses provides a useful experimental framework for future investigations of phytogenic formulations in poultry. Further studies incorporating larger numbers of independent experimental units, quantitative assessment of Salmonella colonization, microbiome profiling, standardized histopathological scoring, intestinal barrier biomarkers, immune response characterization, and controlled measurements of individual HTS intake will be necessary to confirm these findings and to determine their relevance under commercial production conditions. Such investigations will be essential to determine the practical applicability of multicomponent phytogenic formulations as complementary strategies for supporting intestinal health and reducing antimicrobial dependence in poultry production.

## Figures and Tables

**Table 1:** Two-way ANOVA results of microbiological parameters.

Variable	Factor	F	*p*	Partial η^2^ (η^2^p)
Total cultivable aerobic bacteria (TCAB)	Infection status	7.79	0.032	0.565
	nominal HTS concentration	67.5	<0.0001	0.957
	Interaction	4.83	0.056	0.617
*Escherichia coli*	Infection status	304.29	<0.0001	0.981
	nominal HTS concentration	752.07	<0.0001	0.996
	Interaction	909.69	<0.0001	0.997
*Enterobacteriaceae* (ENT)	Infection status	58.35	0.0003	0.907
	nominal HTS concentration	84.07	<0.0001	0.966
	Interaction	10.35	0.011	0.775

**Table 2:** Two-way ANOVA results for histomorphometric parameters.

Variable	Factor	F	*p*	Partial η^2^ (η^2^p)
Mitotic index of intestinal crypts	Infection status	7.71	0.032	0.563
	nominal HTS concentration	3.91	0.082	0.566
	Interaction	18.70	0.003	0.862
Crypt depth	Infection status	368.92	<0.0001	0.984
	nominal HTS concentration	101.30	0.0002	0.971
	Interaction	46.79	0.0002	0.94
Villi thichness	Infection status	186.91	<0.0001	0.969
	nominal HTS concentration	123.24	<0.0001	0.976
	Interaction	5.20	0.049	0.634
Tunica muscularis thickness	Infection status	1.99	0.208	0.249
	nominal HTS concentration	3.95	0.081	0.568
	Interaction	10.09	0.012	0.771

## Data Availability

The original contributions presented in this study are included in the article/Supplementary Materials. Further inquiries can be directed to the corresponding authors.

## Supplementary Materials

The following supporting information can be downloaded at https://www.mdpi.com/article/10.3390/life16081341/s1: Table S1 Cultivable bacterial populations (CFU/g cecal content) in Ross 308 broiler chickens from the experimental groups (L1–L6); Table S2. Quantitative microbiological parameters (log10 CFU/g) in experimental broiler groups (L1–L6); Table S3. Tukey HSD pairwise comparisons for total cultivable aerobic bacteria (TCAB); Table S4. Tukey HSD pairwise comparisons for Escherichia coli (log10 CFU/g); Table S5. Tukey HSD pairwise comparisons for Enterobacteriaceae (ENT); Table S6. Percentage and logarithmic reduction in selected cultivable bacterial populations; Table S7. Measurements of the morphohistological parameters; Table S8. Tukey HSD pairwise comparisons for mitotic index of intestinal crypts; Table S9. Tukey HSD pairwise comparisons for intestinal crypt depth (µm); Table S10. Tukey HSD pairwise comparisons for intestinal villi thickness (µm); Table S11. Tukey HSD pairwise comparisons for tunica muscularis thickness (µm).

## Abbreviations

The following abbreviations are used in this manuscript:

AMR	Antimicrobial resistance
BPW	Buffered Peptone Water
CFU	Colony-forming units
DAB	3,3′-Diaminobenzidine
ENT	Enterobacteriaceae
H&E	Hematoxylin and eosin
HSD	Honestly significant difference
HTS	Herba Top Salmonella
IHC	Immunohistochemistry
log_10_	Logarithm to base 10
MKTTn	Müller–Kauffmann tetrathionate-novobiocin broth
PBS	Phosphate-buffered saline
RVS	Rappaport–Vassiliadis broth
SD	Standard deviation
TCAB	Total cultivable aerobic bacteria
η^2^p	Partial eta squared
