# Supplementary material for: Effects of a Phytogenic Water Supplement on Selected Cultivable Bacterial Populations and Intestinal Histomorphology in Ross 308 Broiler Chickens Challenged with Salmonella Typhimurium
Source: Life (Basel). 2026 Aug 16;16(8):1341. doi: 10.3390/life16081341 (PMC13514616; doi:10.3390/life16081341)
Supplement: Supplementary file 1 [file life-16-01341-s001.zip › life-4444514-supplementary.pdf]

Table S1. Cultivable bacterial populations (CFU/g cecal content) in Ross 308 broiler chickens from the experimental groups (L1–L6).

	TCAB ×10^4 CFU/g	*E. coli* ×10^3 CFU/g	ENT ×10^4 CFU/g
**L1**	86264 ± 419.73	34618.7± 2925	43275.1 ± 1848.2
**L2**	36692.3 ± 5116.7	12187.3 ± 1268.4	28029.3 ± 82.73
**L3**	51311.9 ± 35.78	2819.3 ± 24.19	49136 ± 4627.7
**L4**	45866.7 ± 6025.3	4602.7 ± 109.9	37931 ± 3060.3
**L5**	68693.7 ± 6273.5	40853.5 ± 1911	51733.4 ± 2518.7
**L6**	79695.8 ± 881.9	40937.4 ± 1921.9	66405.9 ± 625.5

Legend: **TCAB** (total number of cultivable aerobic bacteria), *E. coli* (total number of *Escherichia coli*), *ENT* (total number of *Enterobacteriaceae*)

Table S2. Quantitative microbiological parameters (log_{10} CFU/g) in experimental broiler groups (L1–L6).

	TCAB (log_{10} CFU/g)	*E. coli* (log_{10} CFU/g)	ENT (log_{10} CFU/g)
**L1**	8.94 ± 0.002	7.54 ± 0.037	8.64 ± 0.02
**L2**	8.56 ± 0.06	7.09 ± 0.045	8.45 ± 0.001
**L3**	8.72 ± 0.001	6.45 ± 0.004	8.69 ± 0.04
**L4**	8.66 ± 0.056	6.66 ± 0.01	8.58 ± 0.04
**L5**	8.84 ± 0.04	7.61 ± 0.02	8.71 ± 0.02
**L6**	8.90 ± 0.005	7.61 ± 0.02	8.82 ± 0.004

Legend: **TCAB** (total number of cultivable aerobic bacteria), *E. coli* (total number of *Escherichia coli*), *ENT* (total number of *Enterobacteriaceae*)

Table S3. Tukey HSD pairwise comparisons for total cultivable aerobic bacteria (TCAB)

Comparison	Mean difference (log_{10} CFU/g)	95% CI	Adjusted *P*-value
L1 vs L2	0.373	0.224–0.522	0.0005
**L1 vs L3**	0.217	0.068–0.366	0.009
**L1 vs L4**	0.276	0.127–0.425	0.003
**L1 vs L5**	0.100	-0.050–0.249	0.215
**L1 vs L6**	0.034	-0.115–0.183	0.931
**L2 vs L3**	0.156	0.007–0.305	0.041
**L2 vs L4**	0.098	-0.052–0.247	0.228
**L2 vs L5**	0.274	0.125–0.423	0.003
**L2 vs L6**	0.339	0.190–0.488	0.0008
**L3 vs L4**	0.059	-0.091–0.208	0.645
**L3 vs L5**	0.118	-0.032–0.267	0.126
**L3 vs L6**	0.183	0.034–0.332	0.02
**L4 vs L5**	0.176	0.027–0.325	0.024
**L4 vs L6**	0.242	0.093–0.391	0.005
**L5 vs L6**	0.066	-0.084–0.215	0.551

Table S4. Tukey HSD pairwise comparisons for Escherichia coli (log_{10} CFU/g)

Comparison	Mean difference (log_{10} CFU/g)	95% CI	Adjusted *P*-value
**L1 vs L2**	0.454	0.347–0.561	<0.0001
**L1 vs L3**	1.089	0.982–1.195	<0.0001
**L1 vs L4**	0.876	0.769–0.983	<0.0001
**L1 vs L5**	0.072	-0.035–0.179	0.209
**L1 vs L6**	0.073	-0.034–0.179	0.205
**L2 vs L3**	0.63	0.528–0.741	<0.0001
**L2 vs L4**	0.422	0.315–0.529	<0.0001
**L2 vs L5**	0.526	0.419–0.633	<0.0001
**L2 vs L6**	0.527	0.42–0.633	<0.0001
**L3 vs L4**	0.213	0.106–0.32	0.002
**L3 vs L5**	1.161	1.054–1.267	<0.0001
**L3 vs L6**	1.161	1.054–1.268	<0.0001
**L4 vs L5**	0.948	0.841–1.055	<0.0001
**L4 vs L6**	0.949	0.842–1.055	<0.0001
**L5 vs L6**	0.0005	-0.106–0.107	1

Table S5. Tukey HSD pairwise comparisons for Enterobacteriaceae (ENT).

Comparison	Mean difference (log_{10} CFU/g)	95% CI	Adjusted *P*-value
**L1 vs L2**	0.188	0.086–0.29	0.003
**L1 vs L3**	0.054	-0.048–0.156	0.391
**L1 vs L4**	0.058	-0.044–0.160	0.332
**L1 vs L5**	0.078	-0.025–0.18	0.143
**L1 vs L6**	0.186	0.084–0.288	0.003
**L2 vs L3**	0.242	0.14–0.344	0.0006
**L2 vs L4**	0.13	0.023–0.232	0.017
**L2 vs L5**	0.266	0.163–0.368	0.0004
**L2 vs L6**	0.374	0.272–0.476	<0.0001
**L3 vs L4**	0.112	0.01–0.214	0.034
**L3 vs L5**	0.024	-0.079–0.126	0.929
**L3 vs L6**	0.132	0.03–0.234	0.016
**L4 vs L5**	0.136	0.033–0.238	0.014
**L4 vs L6**	0.244	0.142–0.346	0.0006
**L5 vs L6**	0.109	0.006–0.211	0.039

Table S6. Percentage and logarithmic reduction of selected cultivable bacterial populations

Parameter	L2 vs L1	L3 vs L1	L4 vs L6	L5 vs L6
**TCAB**	57,5%<br>0,37 log_{10} CFU/g	40,5%<br>0,23 log_{10} CFU/g	42,4%<br>0,24 log_{10} CFU/g	13,8%<br>0,06 log_{10} CFU/g
E. coli	64,8%<br>0,45 log_{10} CFU/g	91,9%<br>1,09 log_{10} CFU/g	88,8%<br>0,95 log_{10} CFU/g	22,1%<br>0,11 log_{10} CFU/g
ENT	35,2%<br>0,19 log_{10} CFU/g	0	42,9%<br>0,24 log_{10} CFU/g	22,1%<br>0,11 log_{10} CFU/g

Legend: **TCAB** (total number of cultivable aerobic bacteria), *E. coli* (total number of *Escherichia coli*), *ENT* (total number of *Enterobacteriaceae*)

Table S7. Measurements of the morphohistological parameters

	Mitotic index of intestinal crypt	Intestinal villi thickness (μm)	*Tunica muscularis* thickness (μm)	Intestinal crypt depth (μm)
**L1**	0.56 ± 0.02	128.73 ± 3.35	97.01± 9.54	153.05 ± 0.4
**L2**	0.74 ± 0.03	134.41 ± 0.69	122.43 ± 7.38	247.26 ± 10.3
**L3**	0.88 ± 0.06	115.27 ± 1.37	104.74 ± 2.78	252.47 ± 10.11
**L4**	0.7 ± 0.07	122.18 ± 0.82	102.26 ± 10.62	282.59 ± 5.05
**L5**	0.53 ± 0.08	106.23 ± 0.89	131 ± 7.39	319.95 ± 7.28
**L6**	0.68 ± 0.06	112.48 ± 0.03	109.24 ± 4.06	282.24 ± 2.21

Table S8. Tukey HSD pairwise comparisons for mitotic index of intestinal crypts

Comparison	Mean Difference	95% CI	Adjusted *P*-value
**L1 vs L2**	0.18	-0.038–0.398	0.107
**L1 vs L3**	0.32	0.102–0.538	0.008
**L1 vs L4**	0.141	-0.077–0.359	0.237
**L1 vs L5**	0.026	-0.192–0.244	0.996
**L1 vs L6**	0.121	-0.097–0.339	0.352
**L2 vs L3**	0.14	-0.078–0.358	0.242
**L2 vs L4**	0.039	-0.179–0.257	0.974
**L2 vs L5**	0.206	-0.012–0.424	0.064
**L2 vs L6**	0.059	-0.159–0.277	0.875
**L3 vs L4**	0.179	-0.039–0.397	0.109
**L3 vs L5**	0.346	0.128–0.564	0.006
**L3 vs L6**	0.199	-0.019–0.417	0.073
**L4 vs L5**	0.167	-0.051–0.385	0.14
**L4 vs L6**	0.02	-0.198–0.238	0.999
**L5 vs L6**	0.147	-0.071–0.365	0.21

Table S9. Tukey HSD pairwise comparisons for intestinal crypt depth (μm)

Comparison	Mean Difference (μm)	95% CI	Adjusted *P*-value
**L1 vs L2**	94.22	66.46–121.97	<0.0001
**L1 vs L3**	99.43	71.67–127.18	<0.0001
**L1 vs L4**	129.55	101.79–157.30	<0.0001
**L1 vs L5**	166.91	139.15–194.66	<0.0001
**L1 vs L6**	129.2	101.44–156.95	<0.0001
**L2 vs L3**	5.21	-22.54–32.96	0.968
**L2 vs L4**	35.33	7.58–63.08	0.017
**L2 vs L5**	72.69	44.94–100.44	0.0004
**L2 vs L6**	34.98	7.23–62.73	0.018

**L3 vs L4**	30.12	2.37–57.87	0.035
**L3 vs L5**	67.48	39.73–95.23	0.0006
**L3 vs L6**	29.77	2.02–57.52	0.037
**L4 vs L5**	37.36	9.61–65.11	0.013
**L4 vs L6**	0.35	-27.40–28.10	1
**L5 vs L6**	37.71	9.96–65.46	0.012

Table S10. Tukey HSD pairwise comparisons for intestinal villus thickness (μm)

Comparison	Mean Difference (μm)	95% CI	Adjusted *P*-value
**L1 vs L2**	5.68	-0.63–11.99	0.077
**L1 vs L3**	13.46	7.15–19.77	0.001
**L1 vs L4**	6.55	0.24–12.86	0.043
**L1 vs L5**	22.50	16.19–28.81	<0.0001
**L1 vs L6**	16.25	9.94–22.56	0.0004
**L2 vs L3**	19.14	12.83–25.45	0.0002
**L2 vs L4**	12.23	5.92–18.54	0.002
**L2 vs L5**	28.18	21.87–34.49	<0.0001
**L2 vs L6**	21.93	15.62–28.24	<0.0001
**L3 vs L4**	6.91	0.60–13.22	0.034
**L3 vs L5**	9.04	2.73–15.35	0.009
**L3 vs L6**	2.79	-3.52–9.10	0.545
**L4 vs L5**	15.95	9.64–22.26	0.0005
**L4 vs L6**	9.70	3.39–16.01	0.007
**L5 vs L6**	6.25	-0.06–12.56	0.052

Table S11. Tukey HSD pairwise comparisons for *tunica muscularis* thickness (μm)

Comparison	Mean Difference (μm)	95% CI	Adjusted *P*-value
**L1 vs L2**	25.41	-4.42–55.25	0.095
**L1 vs L3**	7.73	-22.11–37.56	0.892
**L1 vs L4**	5.25	-24.59–35.08	0.975
**L1 vs L5**	33.99	4.15–63.82	0.028
**L1 vs L6**	12.23	-17.61–42.06	0.61
**L2 vs L3**	17.69	-12.15–47.52	0.299
**L2 vs L4**	20.16	-9.67–50	0.208
**L2 vs L5**	8.57	-21.26–38.41	0.848
**L2 vs L6**	13.19	-16.65–43.02	0.546
**L3 vs L4**	2.48	-27.36–32.31	0.999
**L3 vs L5**	26.26	-3.58–56.1	0.084
**L3 vs L6**	4.5	-25.34–34.34	0.987
**L4 vs L5**	28.74	-1.10–58.57	0.059
**L4 vs L6**	6.98	-22.86–36.81	0.924
**L5 vs L6**	21.76	-8.08–51.6	0.164
